# Application of Aspiration-Assisted Percutaneous Venous Removal of Right Atrial Thrombus in a Critically Ill Patient: A Case Study and Clinical Management Overview

**DOI:** 10.7759/cureus.28852

**Published:** 2022-09-06

**Authors:** Khan Alam, Gehan A Pendlebury, Peter Oro, James McAlister, Tahreem Hashmi, Hasnan M Ijaz, Ali Chaudhry, Kevin Ergle

**Affiliations:** 1 Internal Medicine, Westside Regional Medical Center, Plantation, USA; 2 Dermatology, Nova Southeastern University Dr. Kiran C. Patel College of Osteopathic Medicine, Davie, USA; 3 Osteopathic Medicine, A.T. Still University, Mesa, USA; 4 Internal Medicine, Nova Southeastern University, Davie, USA; 5 Osteopathic Medicine, Nova Southeastern University Dr. Kiran C. Patel College of Osteopathic Medicine, Fort Lauderdale, USA; 6 Internal Medicine, Critical Care, Northwest Medical Center, Margate, USA; 7 Internal Medicine, Cardiology, Westside Regional Medical Center, Plantation, USA

**Keywords:** anti-coagulation, end-stage renal disease, percutaneous cardiac intervention, hemodialysis, right atrial bypass, catheter-related atrial thrombus, vacuum-assisted thrombectomy, right atrial thrombus

## Abstract

We herein present a complex case of a 50-year-old female with catheter-related atrial thrombus (CRAT). This patient with end-stage renal disease on hemodialysis presented with angioedema leading to respiratory failure. She was subsequently intubated, and the pre-procedural course was complicated by a cardio-respiratory arrest, and anoxic brain injury. The patient’s hemodialysis catheter placement in the superior vena cava (SVC) potentially correlated with the development of the right atrial thrombus. The patient was treated percutaneously as she presented with complex morbidities. The mass was successfully removed via aspiration-assisted percutaneous right heart bypass, a procedure that utilizes a vacuum system to remove thrombi. Post-procedure, the patient remained stable and continued supervised care.

## Introduction

In the United States, the use of central venous catheters (CVC) has become ubiquitous among critically ill patients in the intensive care unit (ICU) [1]. Reported rates of CVC usage range from 13% to 91% for ICU patients [2]. Methods available to reduce complications include catheter designs, standardization of insertion, use of ultrasound guidance, and improvements in central line care. Despite all precaution measures, complications such as central vein thrombosis, catheter placement failure, pneumothorax, and arterial puncture continue to be reported [3]. Catheter-related right atrial thrombosis (CRAT), a specific complication of CVC placement, carries a poor prognosis [4].

CRAT has an estimated incidence of 5.4%-12.5% in vivo and up to 32% among autopsied patients with central catheter in place [4]. The incidence of CRAT among patients with end-stage renal disease and malignancies is estimated to be 5.4% and 9%-18%, respectively [4]. However, evidence-based management guidelines for CRAT in patients with end-stage renal disease (ESRD) are sparse in the literature. We herein present a case of a patient with a history of severe ESRD on hemodialysis who developed CRAT following CVC placement and was successfully treated with vacuum-based removal of the right atrial mass.

## Case presentation

Learning objectives

**Figure 1:**
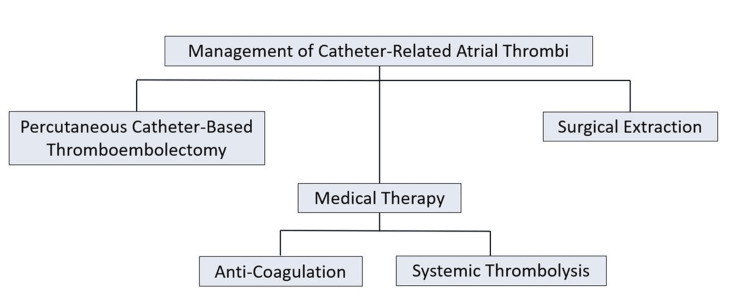
Overview of CRAT management options. CRAT: Catheter-related atrial thrombus

The clinical case serves to provide comprehensive information regarding the clinical presentation, diagnosis, and management of catheter-related right atrial thrombosis. Secondly, the case report serves to provide a further understanding of various treatment modalities for catheter-related right atrial thrombosis. Additionally, this unique case aims to illustrate a clinical comparison pertaining to the advantages and disadvantages of vacuum-based catheterization therapy, particularly in critically ill patients with catheter-related right atrial thrombosis. Figure 1 provides a pragmatic overview of CRAT management options to guide clinical decision-making.

Case presentation

The patient is a 50-year-old female who presented to the emergency department (ED) with a chief complaint of swelling in the tongue and lips with associated shortness of breath after taking nifedipine and hydralazine for the first time. The patient’s medical history includes angioedema secondary to lisinopril, end-stage renal disease (ESRD) on hemodialysis, hypertension, type 2 diabetes mellitus, and diabetic nephropathy. The patient was on multiple antihypertensive medications including hydralazine, nifedipine, losartan, and lisinopril. On arrival at the ED, her respiratory status deteriorated which required endotracheal intubation and mechanical ventilation to maintain her respiratory rate. The patient was admitted to the ICU and typical angioedema management was initiated including steroids, epinephrine, and antihistamines.

**Figure 2:**
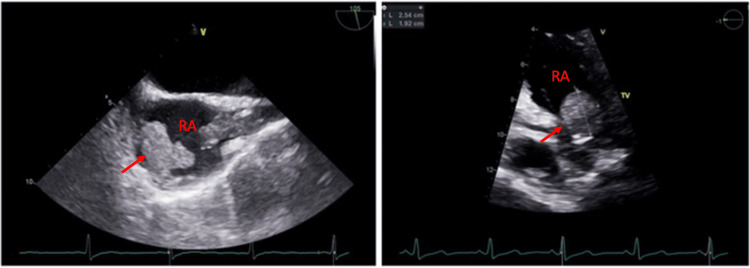
Transesophageal echocardiography showing the right atrial thrombus (red arrows).

After two weeks the patient was transferred to another facility for persistent stridor where she had a tracheostomy performed and a percutaneous endoscopic gastrostomy (PEG) tube placed. Her condition deteriorated due to hypertensive urgency, anemia, and sepsis requiring her to return to our facility. The central catheter was removed and replaced with a right arteriovenous (AV) fistula in the arm to continue dialysis. The creation of the fistula was complicated by the formation of right axillary vein thrombosis. A transesophageal echocardiogram (TEE) was obtained which revealed a greater than 3 cm mass attached to the superior right free wall of the right atrium (Figure 2). The mass was absent in TEE obtained in 2019 and 2020. The placement of the tip of the catheter into the atrial wall confirmed the location of the thrombus. Given her clinical presentation and radiological findings, she was diagnosed with CRAT. Atrial myxoma and endocarditis were considered in the differential diagnosis and were ruled out based on lab findings, physical examination, clinical presentation, and radiological findings.

Management

**Figure 3:**
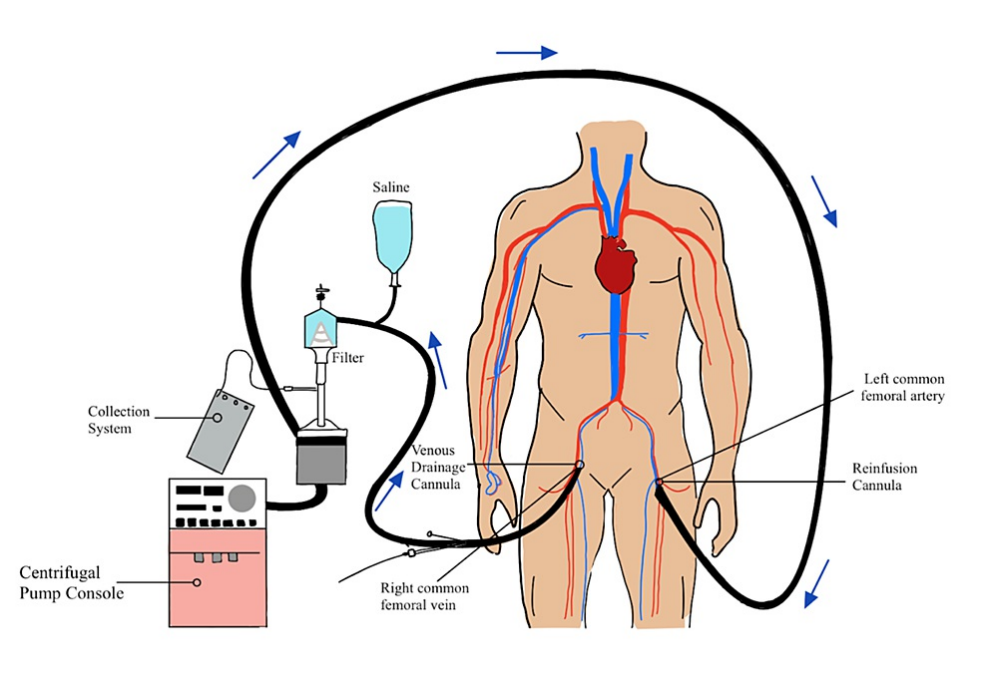
Schematic representation of percutaneous venous removal of right atrial thrombus.

Anticoagulation therapy was deferred due to the large size of the mass and time sensitivity of the patient's critical condition. Likewise, surgical intervention was not indicated due to the high mortality rate and increased risk of complications. As such, percutaneous vacuum-assisted removal (PVAR) was selected as the therapy of choice (Figure 3). This vacuum system utilizes a cannula to facilitate the removal of intravascular thrombi. Four days prior to the procedure, the patient experienced a cardiorespiratory event and required cardiopulmonary resuscitation (CPR) and return of spontaneous circulation (ROSC).

The procedure proceeded as planned. Ultrasound-guided access into the right common femoral vein was obtained. Using ultrasound guidance and micropuncture access, the common femoral vein was cannulated and a 6-French sheath was placed. The patient was given heparin to maintain activating clotting time (ACT) greater than 300 seconds. An Amplatz Super Stiff^TM^ wire was then advanced into the superior vena cava (SVC). The right common femoral vein was dilated with a 26-French dilator and a 26-French Gore Dryseal sheath was placed into the inferior vena cava (IVC). Serial dilation was then performed on the left-sided femoral access site and 18-Fench extracorporeal membrane oxygenation (ECMO) cannula was inserted. A 180-degree vacuum-assisted catheter was then advanced through the Gore Dryseal (over the Amplatz Super Stiff^TM^ wire) and into the cavoatrial junction. Subsequently, the dilator and Amplatz wire were removed.

**Figure 4:**
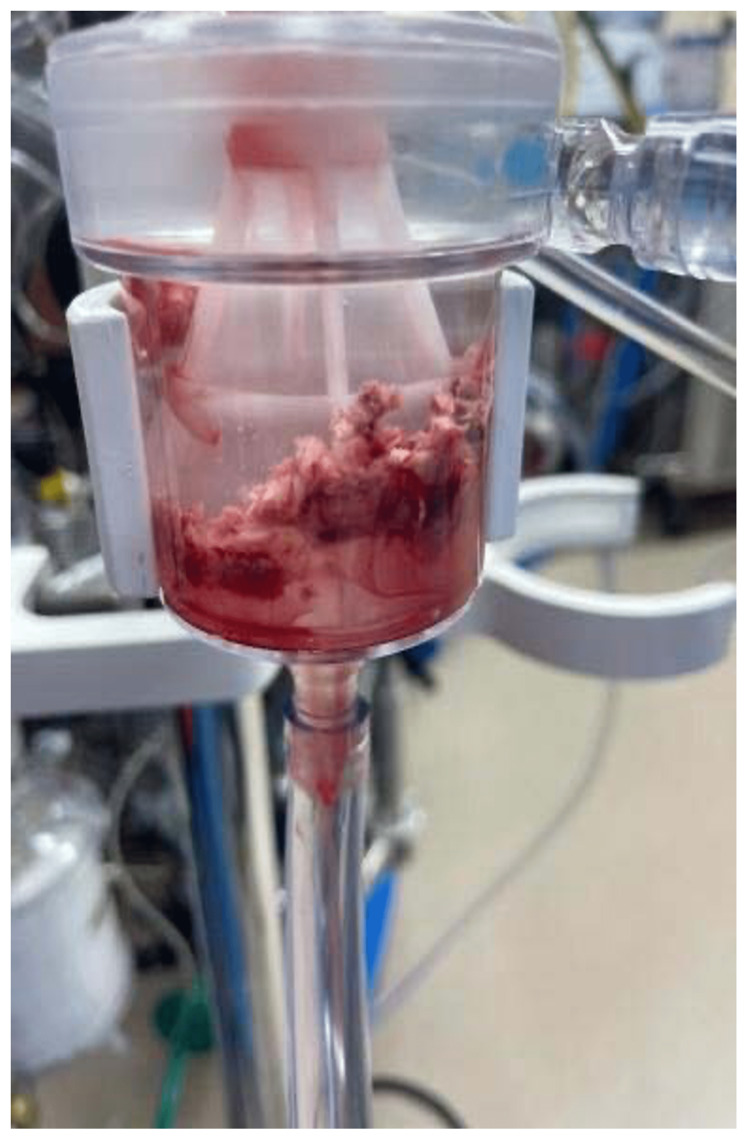
Gross image of extracted thrombus.

A venovenous circuit was established and ECMO was initiated. The catheter removal system was guided via fluoroscopy and TEE. The entire mass, except for a small remnant, was successfully removed (piece by piece) in three passes. The patient tolerated the procedure well. The gross pathology of the thrombus revealed a reddish appearance. The pathology analysis classified the mass as 2.5 x 2.5 x 1.2 cm irregular hemorrhagic soft tissue fragments (Figure 4).

## Discussion

Although effective, central venous catheter placement is associated with adverse sequelae and an increased risk of thrombotic events [4]. Patients with a history of malignancies or ESRD have a higher risk of thrombotic complications [5,6]. It is postulated that repeated mechanical irritation of the atrial wall by the catheter tip activates the coagulation cascade leading to the formation of a thrombus^ ^[7]. In our case, the catheter tip was visualized in the right atrium on imaging, likely triggering the thrombus formation. Likewise, CRAT has a high risk of mortality and other life-threatening complications such as bacteremia, pulmonary embolism, and arrhythmias [6]. Despite the increased morbidity and mortality associated with CVC-related complications, comprehensive management guidelines in critical care settings are sparse, yet needed.

**Figure 5:**
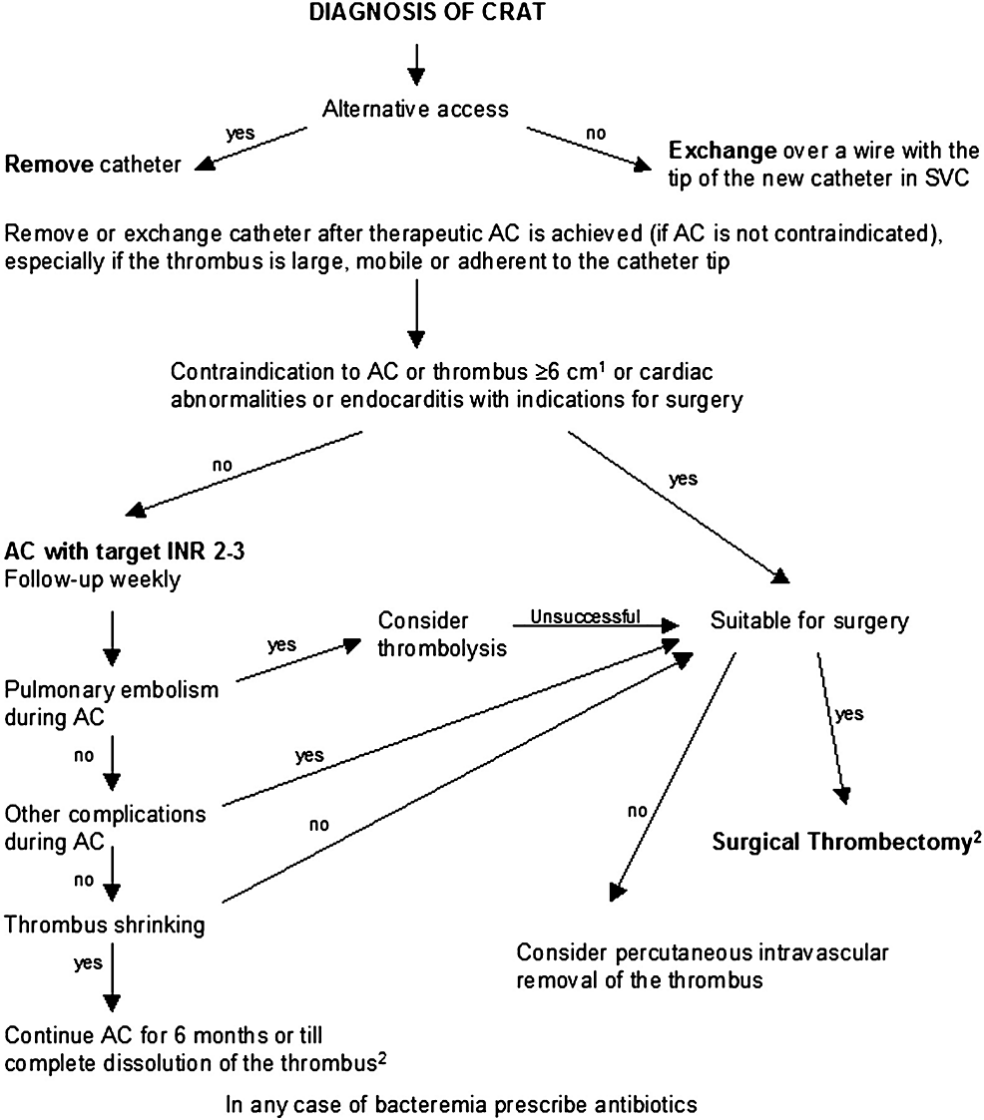
Management algorithm in case of catheter-associated right atrial thrombus (CRAT) in hemodialysis patients. By permission of Oxford University Press [4]. AC: Anticoagulation; SVC: Superior vena cava.

Limited management guidelines exist for such clinical scenarios. A management algorithm for CRAT was developed from a meta-analysis of reported cases [4]. This treatment algorithm includes directive decision-making anticoagulation, thrombolysis, surgical removal, and catheter-directed thrombolysis (Figure 5) [8-10]. Initiation of anticoagulation therapy is recommended following the removal of the catheter. However, comorbidities and contraindications must be assessed prior to starting this treatment. As such, other treatment modalities must be considered [4]. Given its minimally invasive nature, vacuum-based removal of the thrombus is preferred in critically-ill patients and patients with ESRD. This treatment modality has shown a 73% success rate and 87% survival rate following vacuum thrombectomy in critically ill patients [11].

Our patient tolerated the PVAR procedure and recovered well. However, the patient remained in the hospital on mechanical ventilation secondary to angioedema-related and ventilation-dependent respiratory failure. Our unique case (and successful thrombus removal) underscores the utility of PVAR as an underutilized, but highly effective treatment.

## Conclusions

This case outlines the course of a critically-ill patient with a catheter-related right atrial thrombus and serves to draw attention to this severe complication of a common procedure. It offers an example of one approach to management in this patient population and adds to the growing body of evidence supporting the use of vacuum-assisted catheter thrombectomy. Aspiration-assisted percutaneous right heart bypass may successfully prevent further complications in patients in whom coagulation fails. Our unique case highlights a successful application in the management of a critically-ill patient with hemodynamic instability and co-morbidities. Further investigations are necessary to develop a comprehensive management protocol for percutaneous vacuum-assisted thrombectomy for right atrial masses.
